# Genetic and Genomic Tools in Breeding for Resistance to Fusarium Stalk Rot in Maize (*Zea mays* L.)

**DOI:** 10.3390/plants14050819

**Published:** 2025-03-05

**Authors:** Desmond Darko Asiedu, Thomas Miedaner

**Affiliations:** State Plant Breeding Institute, Universität Hohenheim, 70599 Stuttgart, Germany; desmond.asiedu@uni-hohenheim.de

**Keywords:** genomic selection, heritability, inoculation techniques, quantitative scale, quantitative trait loci

## Abstract

Maize (*Zea mays* L.) is the world’s most productive cereal crop, yet it is threatened by several diseases. Among them, Fusarium stalk rot (FSR) causes an average global yield loss of 4.5%. The mycotoxins deoxynivalenol, zearalenone, fumonisins, and moniliformin persist in grain and silage after harvest and pose a risk to human and animal health. This review describes the lifestyle of the fungal pathogens that cause FSR, studies how to optimize resistance evaluation, identifies quantitative trait loci (QTLs) and candidate genes (CGs), and, finally, considers the methods for selecting FSR resistance, especially through genomic selection. To screen maize genotypes for FSR resistance, several artificial inoculation methods have been employed in most studies, including toothpick insertion, ball-bearing pellets, root infection, and the oat kernel method. However, these methods have several limitations in effectively inducing FSR disease infection. Needle injection of inoculum into the stem is recommended, especially when combined with a quantitative or percentage scale because it effectively phenotypes maize populations for FSR resistance. Nine studies with larger populations (≥150 progenies) investigated the genetic architecture of FSR resistance. The inheritance is clearly quantitative. Four major QTLs and several minor QTLs are reported to confer resistance to FSR pathogens, and a few CGs have been identified. Genomic selection is recommended as an effective method for developing routinely FSR-resistant maize, but only two studies have explored this area. An omics analysis (proteomics, transcriptomics, and metabolomics) of the expression of candidate genes should validate their role in FSR resistance, and their use might accelerate selection.

## 1. Introduction

The estimated annual global production of maize (*Zea mays* L.) is about 1.16 billion tons under an area of 203 million hectares [1] and is used for food, bioenergy, feeding livestock, and industrial raw materials [2,3]. Maize accounts for almost 30% of the caloric intake of people in Sub-Saharan Africa [4]. In North-Western Europe, maize is mostly used as animal feed and preserved as silage. The plant is completely cut off just above the ground and subjected to lactic acid fermentation. In the European Union, 5.8 million hectares were harvested as green maize in 2024, representing 246.7 million tons. Another 8.9 million hectares were used for grain maize [5]. Production and quality of both maize varieties are often limited by fungal pathogens, and among the fungal diseases in maize, stalk rot disease is one of the most complex, destructive soil-borne diseases. The composition of the fungal population in stalk rot is highly variable, caused by precipitation, temperature, and humidity, and characterized by a dynamic fluctuation [6,7]. Common diseases affecting maize stalks include Fusarium stalk rot (FSR), which is triggered by various *Fusarium* species ([7,8], rf. to next chapter). Charcoal stalk rot (CSR) stems from the pathogen *Macrophomina phaseolina*, while Diplodia stalk rot (DSR) is attributed to *Diplodia maydis*. Additionally, Pythium stalk rot (PSR) is linked to *Pythium aristosporum*, and Anthracnose stalk rot (ASR) is caused by *Collectotrichum graminicola*. The mycotoxins deoxynivalenol (DON), zearalenone (ZON), fumonisins (FUM), and moniliformin (MON) produced by different *Fusarium* pathogens are known to affect human and animal health [9,10,11,12,13].

FSR is a systemic disease of maize, which attacks the roots and progresses to the aerial parts post-flowering. Silage maize may contain mycotoxins from ear and stalk rot, but for animal feeding, only their fate during ensiling is important [14]. Surveys of maize silage in the US and France have shown that *Fusarium* pathogen populations tend to decrease due to acidic and anaerobic conditions during ensiling [15,16]. However, this process does not eliminate already present mycotoxins [17,18]. The *Fusarium* pathogen can progress to the ear, contaminating the cobs with mycotoxins, and can infect the cobs through the silk, particularly during the early stages of female flowering. Despite this, the outer layers of the cob, reinforced by lignin and silicates, become impenetrable to *Fusarium* hyphae at later growth stages [19]. The rare penetration of intact developing or mature kernels is likely due to the thickness of the pericarp, its wax layer, cell wall phenolic acids, and flavonoid pigments [20,21,22,23]. *Fusarium verticillioides* can also affect the ear by developing an endophyte-like systemic growth within the maize plant [24]. FSR predominantly spreads across internodes. This impedes the water and nutrient transport within the stalk and can lead to smaller grains in the cob and even drying out of total plants. During heavy rain or thunderstorms, the entire crop might lodge, causing significant yield reduction. The increasing number and strength of wind events due to climate change in some maize-growing regions exacerbate this lodging issue caused by the FSR in maize [25]. *Fusarium* species can spread by infected plant residues from the soil within the plant stand and by seeds to the next generation. Moreover, the European corn borer (*Ostrinia nubibalis* Hübner, Lepidoptera: Crambidae) creates wounds on maize stalks, facilitating the infection and accelerating its spread. Infected stalks exhibit a pinkish-to-reddish discoloration of the pith and vascular strands [26].

Globally, stalk rot disease causes varying degrees of yield loss across different geographical locations. In North-West Europe, the average yield losses amount to 4.5%, while in Sub-Saharan Africa it stands at 3.28% [27]. In North America, maize yield losses typically range from 1 to 5% annually with stalk rot ranking second to tar spot disease [28]. Contrastingly, in China, stalk rot leads to an average of 10% reduction in yield, with more severe cases resulting in reductions ranging from 30 to 50% [29]. India experiences an annual yield reduction of 13 to 40% due to this disease [30].

Currently, the management strategy for FSR disease includes the application of chemical fungicides, biocontrol agents, crop rotation, potassium fertilizer, and breeding. Fungicides like kresoximethyl have been utilized to combat stalk rot [31]. Agronomic practices such as tillage to bury crop residues post-harvest, incorporating non-host plants into crop rotation, and appropriate water management during anthesis have also been employed [32]. Potassium fertilizers are known to reduce stalk rot severity, while nitrogen fertilizers may exacerbate it, but this claim lacks substantial support in the literature [33]. Biocontrol management approaches, particularly utilizing biological antagonists like various strains of *Trichoderma* spp. (*Trichoderma harzianum*, *T. asperellum*, *T. viride*), *Bacillus* spp. (*Bacillus cereus* sensu lato strain B25, *Bacillus methylotrophicus*), and *Pseudomonas* spp., have been adopted by researchers [34,35,36,37,38,39]. Biosurfactants such as rhamnolipid have also been used to manage FSR [40]. Maize stalk rot disease was managed via sprays by combining a botanical (neem oil) with *Trichoderma viride* [41]. Despite these efforts, FSR remains inadequately controlled, and some of the chemicals are not environmentally friendly.

The most efficient and preferred approach to controlling stalk rot infections and reducing mycotoxin levels is through the deployment of host resistance [42]. Achieving genetic gain is, however, hindered by large genotype–environment interaction, as shown by multi-locational field trials, differing pathogen populations, reduced trait heritability, a cumbersome inoculation procedure, and missing effective resistance sources. This review aims to (i) elucidate the lifestyle of *Fusarium* pathogens causing FSR disease in different geographical regions, (ii) uncover effective methods for testing maize genotypes to *Fusarium* spp. causing FSR, (iii) unravel phenotypic and genomic approaches employed in identifying quantitative trait loci (QTLs) and candidate genes (CGs) against *Fusarium* stalk rot, and finally, (iv) explore breeding strategies for improving FSR resistance through genomic selection.

## 2. *Fusarium* Pathogens Causing Stalk Rot

*Fusarium* species, which cause FSR, belong either to the *Fusarium graminearum* species complex (FGSC) or the *Fusarium fujikuroi* species complex (FFSC), while some species, such as *Fusarium culmorum*, remain phylogenetically distinct from both of these complexes [43]. The FGSC is taxonomically grouped into 16 species [43,44]. Members of FGSC display different levels of aggressiveness to stalk rot in different geographical locations and they attack a broad range of cereal crops, including maize, wheat, rye, rice, and barley. Among the diverse species of FGSC, *F. graminearum sensu strictu* Schwabe (telemorph *Gibberella zeae*) is considered the most ubiquitous pathogen of maize in Europe and North America [45,46]. Additional species of FGSC-causing FSR in maize include *F. boothii*, *F. meridionale*, *F. cortaderiae*, and *F. austroamericanum,* which are found in South Africa and South America [47,48,49]. The FFSC includes more than 60 phylogenetic species [50] of which *F. equiseti*, *F. proliferatum*, *F. verticillioides*, *F. temperatum*, and *F. subglutinans* have been reported to produce stalk rot in Switzerland, Germany [7,8,45], France, Poland, Belgium [51,52,53], and China [29].

These pathogens also exploit weeds as alternative hosts, serving as primary sources of infection, and also tend to persist in residues from previous crops [54]. Their life cycle includes several stages; they survive harsh environmental conditions, such as the winter season, either as a sexual stage called perithecia or as mycelia, both in crop residues. An example of this lifestyle is *F. graminearum*, a homothallic and self-fertile fungus known as *Gibberella zeae* in its sexual stage. During the sexual phase, it releases ascospores into the atmosphere with remarkable force [54,55]. Furthermore, asexual spores (macroconidia) are released from crop debris and infected plant tissues by the pathogen, and they are primarily dispersed through rain splash within the plant stand [54]. Ascospores and macroconidia are the major sources of infection. Infection primarily thrives under conditions of high relative humidity (>80%), and the fungus grows best at 20 °C [56].

However, *F. graminearum* (Phylum: Ascomycota), the predominant pathogen in Central Europe not only decays maize stalks (and ears) but also produces B-type trichothecenes such as nivalenol or deoxynivalenol, along with their acetylated derivatives, similar to many other *Fusarium* pathogens. In addition, *F. graminearum* is hemibiotrophic in nature like most *Fusaria*, where it begins with a short biotrophic lifestyle (derives nutrients during intracellular growth of hyphae in the initial stages of infection without symptoms) followed by a rapid necrotrophic infection (massive cell death and feeds on dead tissue in the later stages) [57].

*F. verticillioides* also occurs in Northwest and Central Europe during warm summers but is the most commonly isolated fungus of maize in warmer regions like southern France, Spain, Italy, and Brazil [58,59]. FSR caused by *F. verticillioides* may, in the worst case, result in a 30 to 50% reduction in maize yield [60], and it also behaves hemibiotrophically, persisting particularly around 27 °C and even producing spores on stalk residues for FSR infection at 45 °C [61]. In favorable conditions, this soil-borne fungus aggressively multiplies in roots and mesocotyls and then spreads through maize stems via the vasculature, inducing stalk rot. It can persist throughout the plant’s life cycle, even when originating from the seed. Seeds contaminated by *F. verticillioides* may exhibit asymptomatic seedling infection. The fungus then behaves as an endophyte and can remain in maize residue for up to about 21 months [62].

*F. subglutinans* primarily thrives in temperate regions and is closely related to *F. temperatum* due to the production of the mycotoxin fumonisin B1. Despite their genetic relatedness, Belgian fields exhibited a remarkably high *F. temperatum* to *F. subglutinans* ratio (around 30:1), indicating *F. temperatum* superiority over its sister species [63].

In several European countries, approximately 17% of maize stalk rot infections are from *F. temperatum*, whereas only 2% are attributed to *F. subglutinans* [45,52]. The former displayed a higher level of aggressiveness compared to the latter in their assessment of pathogen aggressiveness on Korean maize cultivars [64]. *F. subglutinans* tend to inhabit primarily warmer and drier regions such as Italy, Slovakia, and Serbia [65]. In contrast, *F. temperatum* is more commonly found in moderate to cooler climates, typically with mean temperatures of 18 °C or lower, like in Poland [51] and Germany [45]. *F. temperatum*, exhibits a disease cycle in maize that is divided into two stages, starting with a biotrophic phase where microconidia germinate on the root surface, followed by hyphal proliferation surrounding the root, ultimately advancing to the xylem vessels [66].

In a study by Qiu et al. [67], *Fusarium* isolates from 260 stalk and kernel samples across 42 districts in northeastern China, the largest maize-growing region in China, were analyzed. *F. graminearum* emerged as the most prevalent species, comprising 67.4% of the isolates, while all other *Fusarium* spp. including *F. meridionale* and *F. boothii,* occurred at low frequencies. In Brazil, *F. meridionale* dominates maize ears and stalks [68]. A two-year survey of naturally infected maize stalk samples from 190 sites in Germany revealed *F. graminearum* (65%) as the most dominant species causing FSR, followed by *F. equiseti* (22%), *F. culmorum* (19%), and *F. temperatum* (17%) as the most prevalent *Fusarium* species [8]. This was in agreement with an aggressiveness study by Asiedu et al. [69], who pointed out *F. graminearum* as the most aggressive pathogen in causing stalk rot infections in 22 maize hybrids compared to *F. temperatum* and *F. culmorum*. In addition, when considering Central Europe as a whole, all other *Fusarium* species display reduced disease severities in comparison to *F. graminearum*.

Plants respond to *Fusarium* pathogen invasion by producing mitogen-activated protein kinases (via calcium signaling), generating reactive oxygen species, and depositing callose to strengthen cell walls at infection sites [70]. Nonetheless, most *Fusarium* species utilize cell-wall-degrading enzymes and trichothecene mycotoxins to speed up invasion [57]. Most *Fusarium* spp. use their pathogenic toxins to hinder the plant’s defense response against fungal attack by inhibiting the synthesis of resistance-related proteins [71] and subsequently causing FSR infection.

## 3. Phenotypic and Molecular Approaches for Resistance Improvement

A prerequisite for breeding for FSR resistance is a successful resistance testing system involving reproducible, artificial inoculation of the pathogens and a standardized quantitative recording of symptoms. Both must be applicable to larger maize populations, should have the highest possible precision, be reproducible, and allow for optimal genetic differentiation. The first step is, therefore, to define a uniform system for resistance testing. The heritability of resistance assessment plays a particularly important role in the success of selection. This is maximized by a large genetic variance in the plant populations and a small experimental error. The size of the genotype–environment interaction variance can only be controlled indirectly by the number of environments. Another important variable is the possible influence of agronomic traits on the level of resistance. These can have a negative effect on resistance or, conversely, act as an indirect trait if the relationship is close enough. Finally, classical genetic and QTL analyses can be used to determine the genetic architecture of FSR resistance.

### 3.1. Resistance Testing and Evaluation

Efficient phenotypic screening techniques depend largely on inoculation timing, method, quantitative indicators of disease severity, and workload. In the case of *Fusarium* stalk rot resistance in maize, these key factors are cumbersome to detect. It is crucial to choose the most reliable inoculation technique that results in an adequate level of infection to categorize genotypes into groups based on their disease response combined with a statistically valid disease rating system [72,73].

Natural infection was preferred for selection, as its disease symptoms are different from artificial infection [74,75,76]. However, natural infection can have uneven pathogen distribution, occurrence of different *Fusarium* species, and interactions with other pathogens in the field [77,78,79]. In contrast, artificial inoculation allows a more stable and uniform disease pressure for a reproducible phenotypic performance of genotypes [69,80,81,82].

Several artificial inoculation techniques for FSR infection have been developed (Table 1). Artificial wounding of the stem to introduce inoculum into the plant is predominant. For artificial wounding, either a needle injection or the toothpick method is available (Table 1). In 1948, Young [83] introduced the toothpick method, entailing boiling toothpicks with deionized water for several hours to remove tannins, soaking them in a nutrient-containing medium, autoclaving, and then incubating them with a fungal culture containing *Fusarium* spores in darkness to produce a usable inoculum. Plants are typically inoculated with toothpicks during the tasseling (VT) phase. Prior to introducing the toothpicks, the stalks are punctured by sterilized 1.8 mm diameter nails to create a 1 to 2 cm deep hole into the second or third internode from the soil level [80] to ensure infection. Toothpicks tend to soften due to fungal growth. More recently, inoculum-covered ball bearing (BB) pellets [84] were shot into maize stalks using a gas pistol one week after anthesis. This inoculation approach creates larger wounds via the ball bearings to increase fungal infection. Both approaches cannot quantify the inoculum dose of the pathogens [78,85].

Needle injection entails inoculating the stalk with a hypodermic needle or syringe containing a spore suspension, adjusted to a density ranging from 60,000 to 4 × 10^6^ spores/mL. A 2 cm hole is created with a jabber in the second internode, typically at the VT or silking (R1) stage when FSR is usually starting. At this time, there is a shift in carbohydrate flow from the stalk to the ear, root cell senescence occurs, and maize stem reserves are limited in assimilating, making them more susceptible to stalk rot infection [93]. Each injected stalk is examined for FSR symptoms about 30 to 55 days post-inoculation [88]. A recent study evaluated the effectiveness of inoculation methods with different aggressiveness of *Fusarium* spp. on FSR resistance in maize hybrids [69]. Needle injection displayed the highest stalk rot infection compared to toothpick inoculation or inoculation by mycelium or an inoculum-coated stick. Needle injection proved to be the most effective approach for differentiating genotypic resistance, as it recorded the highest mean internode proportion, a well-established quantitative scale for discriminating maize genotypes for FSR resistance level at physiological maturity. This inoculation method has shown great promise in ranking hybrids based on resistance levels, as it establishes a uniform disease condition with a known amount of inoculum across maize genotypes and environments [78,88,94]. Needle injection has also been efficiently employed by Babu et al. [88] in double haploid (DH) maize lines and is widely used by scientists for resistance breeding in maize [64,76,78,82,94,95].

Root infection encompasses digging a hole in the ground and placing infected maize or wheat kernels in it a small distance (5 to 10 cm) away from the stem of each maize plant at silk emergence, followed by irrigation to maintain soil humidity and boost fungal growth and infection [95]. Inoculum is prepared from sterilized kernels mixed with a slab of *F. graminearum*, obtained from agar culture, and incubated at 25 °C in complete darkness for 15 days. Plants are rated for symptoms of stalk rot infection 1 month post-inoculation. Although the root infection method was initially used by Yang et al. [96] to map FSR resistance genes, root infection has seen limited adoption [91,95] due to its low disease incidence, poor inoculation stability, difficulty in inoculum preparation, and uneven distribution of pathogens in the soil [97].

In the oat kernel inoculation method, oat kernels are surface sterilized in 70% ethanol for 2 min, followed by a 20% bleach solution with Tween 20 for 20 min, then rinsed three times in distilled water. After 12 h of drying, the kernels are placed in 20 mL glass holders and sterilized in a water bath at 60 °C (2 × 5 min), followed by 10 min in dry air at 121 °C. Sterilized oat kernels are mixed with a 2 mL suspension of *F. graminearum* (10^6^ conidia/mL). A 2 mm hole is created in the second internode of the maize stalk 12 days after flowering, and infected oat kernels are placed in it. Holes are sealed with a sticking plaster after inoculation. Plant symptoms are assessed for FSR infection using Colim 4.0 software one-month post-inoculation [92].

Inoculated maize plants are evaluated for FSR severity marked by percentage discoloration at physiological maturity (R6) by visual rating with a qualitative or quantitative ordinal scale or a percentage scale. Percentage scale is rarely used because many scientists found it too labor intensive for large-scale evaluations compared to the qualitative scale, but in reality, FSR resistance is inherited by polygenes and phenotypically follows a quantitative distribution, and as such, a percentage scale is advantageous [98]. Quantitative ordinal scales are composed of ordered categories that are derived from an underlying metric scale (such as the percentage of diseased stalk area), where the intervals between cutpoints are equally spaced [99]. In practice, quantitative ordinal scales, such as four, five, or six-point rating scales (each covering 25 to 100%) and a nine-point rating scale (covering 12.5%) [64,84,88,90] are usual disease assessment tools in the hands of plant breeders compared to qualitative ordinal scales like 0–9, 1–6, and 1–9 ratings [78,82,86]. Some of the commonly used quantitative ordinal scales include Hooker’s scale (Table 2), whereas a few use the Payak and Sharma scale [100,101]. It is impractical to use qualitative ordinal scales to rate stalk discoloration. Additionally, estimating the means or midpoints of these scales and linking values to biological conclusions from FSR experiments is statistically inappropriate [102], and this violates the assumptions of parametric tests of normal distribution. Internode proportion, i.e., the percentage of visible infection covered by mycelium and/or necroses summed over the number of internodes affected in a longitudinally split maize stalk, was reported to be an ideal quantitative scale to discriminate maize genotypes based on FSR disease severity with a high entry-mean heritability (*H*^2^ = 0.90) [69]. Several studies have emphasized the precision of such direct percentage scales in several pathosystems, including FSR [89,103], *Gibberella* ear rot [104,105,106], northern corn leaf blight in maize [107], and oat crown rust [108]. Alternatively, image analysis software is used to provide a detailed assessment of FSR, but it is difficult to apply to large maize populations in the field [92,109].

In summary, the toothpick, ball bearing, and root infection methods lack the ability to control and monitor inoculum dosage effectively. Moreover, oat kernel inoculation is limited by inconsistent and/or low FSR infection rates across genotypes, as fungal colonization may be variable. We recommend needle injection for evaluating quantitative FSR resistance because it is a widely used method for delivering a precise and adjustable spore density of *Fusarium* inoculum. Additionally, it ensures uniform disease pressure across genotypes and enables constant reproducibility of genotypic responses to FSR infection across different locations. As a quantitative trait, the internode proportion of colonized tissue within each internode should be used.

### 3.2. Phenotype-Based Genetic Studies for Resistance to FSR

Classical quantitative genetic studies have established that FSR resistance is a quantitative trait controlled by multiple loci with minor effects [81,89,90,95,110]. Analysis of general combining ability (GCA) effects in half-diallel studies of maize inbreds has also demonstrated that resistance to FSR is primarily driven by additive gene action [111,112]. In some maize genotypes, resistance was associated with one or a few dominant genes [85,96], while other genotypes showed resistance mediated by recessive genes [81]. Also, the moderate heritabilities (Table 3) of FSR resistance indicate that it is controlled by polygenes with minor effects and a high effect of genotype–environment interaction.

A critical consideration in designing a breeding program is the genetic correlation between inbred lines and their (test)crosses for FSR resistance. The presence of additive variance suggests that the covariation should be substantial, thus enabling selection based on doubled haploid (DH) lines. However, this assumption remains unproven. Despite this, a significant correlation (*r* = 0.72, *p* < 0.05) has been observed between general combining ability (GCA) effects and stalk rot scores among twelve inbred lines [111] using an inappropriate quantitative scale. Substantial literature supporting this finding is lacking.

Sources of resistance to Fusarium stalk rot (FSR) have been identified in studies by Song et al. [110], which reported reduced disease severities in the Tuxpeño (TUX) heterotic group, with a mean stalk rot severity of 16% compared to the DTMA maize population, which had a mean stalk rot severity of 46.7% [89]. TUX was derived from both temperate and tropical inbreds.

Generation of doubled haploid (DH) lines by maternal haploid induction is now a standard technique in commercial maize breeding. These DH lines can be multiplied and tested for their performance against FSR and other traits at several locations using a reliable inoculation method, such as needle injection, and an efficient resistance evaluation technique, such as the internode proportion method. Selecting stalk breakage alone as a resistance criterion, without artificial inoculation, is not suitable since breakage can result from either disease susceptibility or the stalk’s mechanical strength, regardless of FSR presence [114]. However, it could serve as an additional trait when disease pressure is sufficiently high. Another option would be to harvest the maize populations about three to four weeks later than normal in order to increase the disease pressure on the stalk. This might also increase the heritability of resistance assessment. When no specific sources of resistance to FSR are available for a special region or gene pool, breeders need to improve their own materials to detect more resistant inbred lines. As the allele frequency of resistance loci may be low in the beginning, reciprocal recurrent selection (RRS) may be beneficial to increase the frequency of useful alleles. A pure phenotypic RRS scheme could be established based on the selection of superior S1 or DH lines after inoculation, which are recombined in the winter nursery abroad and selected again the following year. They can additionally be selected for adaptation traits and other disease resistances. Depending on the status of the population in terms of grain yield and moisture content, a few cycles may suffice before the selected lines are introduced into the commercial line development program.

### 3.3. Heritabilities and Correlations with Agronomic Traits

A crucial parameter for breeding is the entry-mean (broad-sense) heritability of a trait, as it determines the effort required for trait recording and is an important component of selection gain [115]. The lower the heritability, the more locations and years are needed to reliably estimate the genetic value and the lower the selection gain per cycle. The size of broad-sense heritability depends on the number of locations and years and, to some extent, also on population size. FSR disease depends greatly on environmental conditions like temperature and humidity during flowering, affecting fungal infection. To effectively evaluate genotypic differences of maize in response to FSR, it is recommended to test with the most aggressive and prevalent *Fusarium* spp. under optimal conditions. However, weather is highly variable in temperate regions. Broad-sense heritability ranges for FSR between 0.37 to 0.86 (Table 3) and is in most papers closer to 0.5, indicating a high importance of the genotype–environment interaction and/or error variances, which requires phenotyping in many locations/years. Other reasons for the lower estimate are most likely the inaccuracy of the inoculation, unfavorable environmental conditions affecting *Fusarium* pathogen aggressiveness as well as limitations in evaluation procedures, and the lack of true resistance donors.

The incidence of FSR in maize is primarily influenced by the plant’s growth stage [116] and the interaction with biotic and abiotic stresses that predispose the plants to infection, particularly after anthesis when carbohydrate shortages occur [117,118,119]. There is a negative correlation (*r* = −0.56; *p* < 0.01) between grain yield and stalk rot in maize [110]. The presence of stalk rot accelerates the decomposition of soluble sugars, reducing stalk strength and increasing the risk of stalk lodging [114]. Stalk lodging is positively correlated with stalk rot [120] and is influenced by factors such as genotype, plant density, plant morphology, carbohydrate content, and mechanical strength of the stalk, as well as environmental conditions and cultivation practices. While a small angle of lodging has minimal impact on yield, a severe angle significantly reduces yield [121,122].

Stalk breakage had a significant negative correlation with days to silking (*r* = −0.71; *p* < 0.001) [69]. Severe stalk breakage interrupts grain filling in the entire plant due to the death of the plant above the break, leading to yield reductions or even complete crop failure. Fungal hyphae typically block the parenchymatous cells in the vascular bundles of the pith, impairing the transportation of water, nutrients, and soluble carbohydrates from photosynthesis and accelerating stalk senescence. Senescence, along with the degradation of carbohydrates and moisture loss, reduces the stalk’s mechanical strength, leading to lodging after the R6 stage. Moreover, lodged plants create many difficulties with mechanical harvesting.

Enzyme reactions play a crucial role in maize’s resilience against FSR infection by strengthening its defense mechanisms. This is achieved through the fortification of the cell wall, detoxification of reactive oxygen species, and the synthesis of antimicrobial compounds. Increased activity of peroxidase (POD), polyphenol oxidase (PPO), and phenylalanine ammonia lyase (PAL) is strongly associated with improved plant defense against FSR infection. These biochemical processes elicited by these enzymes enhance maize resistance to pathogen attacks.

POD are oxido-reductive enzymes critical to cell wall fortification through the oxidation of phenols, suberization, and lignification of host plant cells, particularly during defense responses against pathogenic agents [123,124]. These enzymes, alongside superoxide dismutase, act as essential reactive oxygen species scavengers in plants. Notably, high levels of POD activity are linked with improved resistance to phytopathogens in cereals [125,126,127,128] and sugarcane [129]. PPO catalyzes the oxidation of polyphenols to quinones, which are antimicrobial compounds, and facilitates the lignification of plant cells during microbial invasion. PPO-mediated reactions involve the oxidation of phenolic compounds to quinones, with molecular oxygen serving as the electron acceptor [130,131]. PAL is a key enzyme in the phenylpropanoid pathway and plays a central role in plant resistance to pathogens, insect attacks, and environmental stresses [132]. In addition, catalase (CAT) catalyzes the decomposition of hydrogen peroxide into water and oxygen, mitigating oxidative damage during plant defense responses.

**Figure 1:**
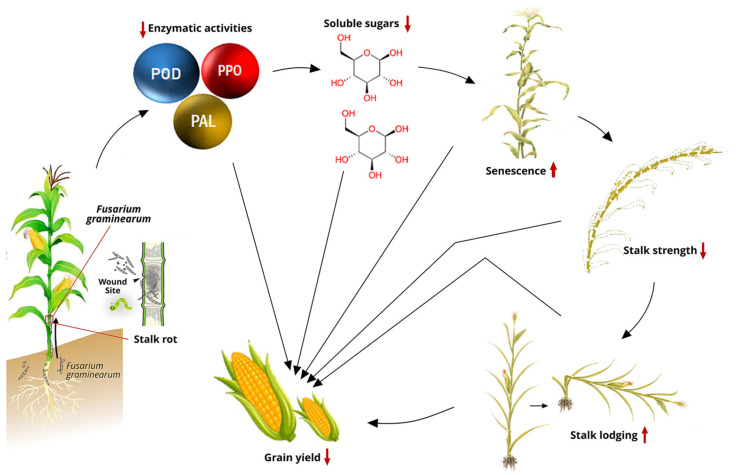
Interrelationships between stalk rot and agronomic traits in a susceptible maize genotype after R6 [114,137,138]. Abbreviations: POD = peroxidase; PPO = polyphenol oxidase; PAL = phenylalanine ammonia lyase.

Maize inbred lines resistant to *Fusarium graminearum* demonstrated significantly higher enzymatic activities of CAT, PAL, PPO, and superoxide dismutase compared to susceptible lines [133]. Additionally, Li et al. [134] observed that PAL and POD activities in resistant maize varieties increased more rapidly than in susceptible varieties during the tasseling stage after inoculation with *F. graminearum*. Clearly, the activities of PAL, POD, and PPO exhibit a positive correlation with resistance to maize stalk rot (Figure 1) [114].

Stalk rot pathogens require energy during infection, primarily using soluble sugars since structural carbohydrates like cellulose and lignin are not easily degradable (Figure 1). The resistance of maize plants to stalk rot is related to the physiological activity and soluble sugar content of the stalk. Higher water and soluble sugar content in maize correlates with higher resistance to stalk rot after infection and can be used as a breeding index for selecting FSR-resistant genotypes. Applying potassium between the V6 and flowering stages can increase the accumulation of structural carbohydrates and enhance stalk strength, thereby reducing FSR [135,136].

Research has also shown a correlation between stalk rot and ear rot in maize and this is linked to the consistent isolation of the same *Fusarium* pathogens, particularly *Fusarium verticillioides*, and FGSC, from stalks, ear, and kernels across various regions [11]. *Fusarium* infection typically starts at the roots, lower stalks, or seedling stage, spreads through the vascular system, and eventually infects the ears. A positive correlation exists between DON accumulation in stalks and lesion areas of maize stalks [42]. The mycotoxin concentration can serve as a phenotypic marker for disease level in high-throughput (but costly) metabolite phenotyping.

### 3.4. QTL Studies and Candidate Genes for FSR Resistance

**Figure 2:**
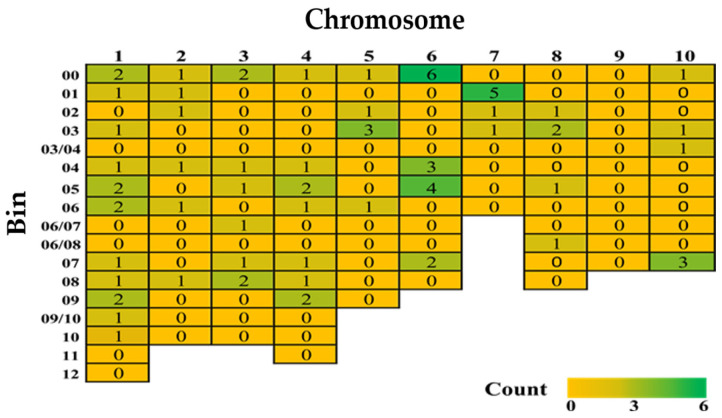
A heatmap of reported quantitative trait loci for Fusarium stalk rot resistance from nine studies in 1993–2024 (for details and references, please refer to Table S1).

The quantitative inheritance of FSR resistance implies that there could be a large array of QTLs responsible. In comparison to Fusarium or Gibberella ear rot resistance, however, only a few QTLs associated with FSR in maize have been identified through linkage and association mapping. The main reason is that till now only nine papers have reported QTLs for FSR resistance (Table S1). A total of 76 QTLs were mapped across the maize genome, covering all chromosomes except chromosome 9 (Figure 2). Among these, 46 minor QTLs were distributed across the genome, while two major QTLs accounted for 36.3% and 34.4% of the phenotypic variance (PVE), respectively (Table 4).

The remaining 28 QTLs did not have reported PVEs (Table S1). Chromosome 6, followed by chromosome 1, contained the highest number of minor QTLs. Genomic bins 6.00 and 7.01 had the highest number of QTLs linked to FSR resistance (Figure 2), while chromosome 1 had minor QTLs across almost all genomic bins (bins 1.00 to 1.09, except 1.11–12). Notably, chromosome 1 is a hotspot harboring several quantitative disease resistances, including Gibberella ear rot, common smut (caused by *Ustilago maydis*), gray leaf spot (caused by *Cercospora zeae-maydis*), southern corn leaf blight (caused by *Bipolaris maydis*, syn. *Helminthosporium maydis*), northern corn leaf blight, and Stewart’s wilt (caused by *Pantoea stewartii*) [139,140,141,142].

In an early study of FSR resistance, Pè et al. [113] discovered five QTLs jointly explaining phenotypic variation (PVE) of 20% on chromosomes 1, 3, 4, 5, and 10 using an F_2:3_ mapping population developed from B89 × 33-17 by the composite interval mapping approach. In another study, three QTLs with resistance to stalk rot had a moderate proportion of explained phenotypic variance from 5.42 to 19.62% via linkage mapping coupled with GWAS within bin 3.02, 4.06/08, and 8.02/03 [86]. QTL analyses of a backcross population from 1145 × Y331 revealed a large effect QTL (*qRfg1*) with a phenotypic variation (PVE) of 36.3% on chromosome 10 and a minor QTL (*qRfg2*) with a PVE of 8.9% on bin 1.09/10 [95]. *qRfg1* was fine mapped to a ~500 kb region and high-density markers have been developed within and around this region to allow for marker-assisted selection (MAS) in FSR resistance breeding programs. A CCT domain-containing gene, *ZmCCT*, was further cloned from the QTL-*qRfg1* region and is known as the causal gene for resistance to stalk rot at this locus [143]. To highlight the importance of the QTL-*qRfg1*, *ZmCCT* haplotype H5 is known to enhance grain yield, FSR resistance, and drought tolerance in maize [144]. The minor QTL-*qRfg2* has been narrowed down to a physical distance of ~300 kb flanked by markers SSRZ319 and CAPSZ459 and is known to increase the FSR resistance by ~12% across backcross generations [145].

Interestingly, QTL-*qPss3* on chromosome 3 is fine-mapped to an interval of ~120 kb and is known to suppress photoperiod sensitivity to high-latitude geographical locations as a result of induction by *ZmCCT9*. When present with *ZmCCT10* in the heterozygous state, it offers FSR resistance without delaying flowering significantly [146]. The potential of QTL-*qPss3* could be explored to ensure synchronous flowering of parental lines in FSR breeding programs. The second major QTL-*Rgsr8.1* with a PVE of 34% was fine-mapped to a 2.04 Mb interval on chromosome 8, flanked by SSR-65 and SNP-25 markers at 164.69 to 166.72 Mb, revealing two putative candidate genes: *Zm953*, an auxin response factor, and *Zm972*, a disease resistance protein, both showing higher expression in resistant plants and likely contributing to FSR resistance [91]. Ma et al. [81] identified another QTL-*qRfg3* (10.7–19.4% PVE) on chromosome 3, which provides recessive resistance to stalk rot, reducing the disease severity index by about 26.6%. Gene pyramiding resistant QTLs like *qRfg1* and *qRfg2* could enhance and stabilize resistance to FSR in maize inbred lines in the future.

Currently, a comprehensive GWAS study in tropical maize with 381 lines from the Tuxpeño heterotic pool and 296 non-Tuxpeño lines found 18 SNPs, which were significantly associated with FSR resistance [110]. Three useful CGs associated with these SNPs include *GRMZM2G122025* (a stress response NST1-like protein), *GRMZM2G046021* (a histone acetyltransferase GNAT/MYST), and *GRMZM5G860810* (a leucine-rich repeat (LRR) protein kinase family protein associated with biotic and abiotic stress response in plants) [110,147,148,149]. Moreover, a similar association study was carried out on a diversity panel of 165 inbred lines of temperate and tropical origin, and this revealed three other putative candidate genes: *GRMZM2G082709*, *GRMZM5G841142*, and *GRMZM2G080041* [86]. The first two CGs were up-regulated in CDMA66 (FSR-resistant inbred) and down-regulated in Huangzao 4 (FSR-susceptible inbred) 12 h and 72 h post-inoculation, respectively, whereas the expression level of *GRMZM2G080041* was only up-regulated in Huangzao 4 12 h post-inoculation of *Fusarium graminearum* after gene expression analysis [86]. These CGs are known to be associated with plant growth and development, stress responses, plant metabolism, transcription and translation regulation, signal transduction, plant resistance, and pathogen identification [150,151,152]. A stable genomic region at ~250 Mb on chromosome 1 (bin 1.08/9) was found to be significantly associated with FSR resistance. CGs on this chromosome region included *GRMZM2G457357*, *GRMZM2G364069*, *GRMZM2G027991*, *GRMZM2G070323,* and *AC213890.4_FG004* [89]. Studies showed that these CGs are involved in abiotic and biotic stress responses, protein regulation, and cellular processes, which are crucial for enhancing plant defense mechanisms against FSR.

In summary, QTL(s) are largely influenced by environmental factors [26,92,153] and the genetic background of the genotypes (e.g., by epistasis). Significant QTL–environment interactions tend to reduce heritability and the effectiveness of selection for FSR resistance traits in large breeding programs. To date, only a few major QTLs have been identified (Table 4), and their variable expression in different genetic backgrounds and environments has not been fully explored. Therefore, it is critical to validate these QTLs before incorporating them into commercial FSR breeding programs to confirm their effectiveness and consistency. Validation should ensure that the identified QTLs reliably confer resistance across various genetic backgrounds and environments, thereby enhancing the accuracy of marker-assisted selection (MAS) in developing resistant FSR maize cultivars. Without validation, QTLs may perform inconsistently, leading to ineffective MAS and genomic selection (GS) procedures, reduced or even missing genetic gains, and significant economic losses due to crop failures.

MAS or MARS afford highly consistent QTLs that are reproducibly expressed in successive cycles. Given that the two major QTLs *qRfg1* and *Rgsr8.1* could be verified in different genetic backgrounds, they could afterward be introduced into the respective heterotic group by marker-assisted selection/backcrossing and selected by markers in subsequent cycles. However, the linked marker(s) may not remain in the same linkage disequilibrium block with the causal gene across generations due to recombination. The likelihood of recombination decreases when the linkage is very close. Therefore, the availability of the target QTLs/genes needs to be demonstrated from time to time by phenotypic assessment of FSR resistance in the field.

MAS has been shown to be successful in selecting for resistance to Fusarium crown rot in wheat, a quantitative trait with a similar complex inheritance pattern to FSR [154]. After two cycles of marker-assisted recurrent selection (MARS) with up to 22 major QTLs, a significant and positive response to selection was found to be related to the original parents and the base population in this study. Whether this procedure is reasonable in a competitive commercial maize breeding program has to be proven. Selecting so many QTLs for only one trait will further fix large parts of the genome in the vicinity of the selected alleles, thus limiting variation for other traits.

Major QTLs with significant effects can segregate like single Mendelian loci in a uniform genetic background, with their genetic effect consistently maintained across generations. In contrast, minor QTLs pose a challenge for accurate phenotypic data collection due to their small genetic contributions and occasional undetectability in advanced mapping populations. Consequently, combining multiple minor QTLs through genomic selection (GS) should be more valuable for plant breeders.

## 4. Genomic-Assisted Selection Strategies

GS should be more advantageous for improving FSR resistance than MAS procedures given the high frequency of small-effect QTLs and their additive inheritance. GS reduces both the cost per breeding cycle and the time needed for improving varieties compared to pure phenotypic selection [155]. The precision of GS for FSR resistance depends on trait heritability, population size, marker density, genotype–environment interaction, prediction models, and the genetic relatedness between training and prediction sets.

**Figure 3:**
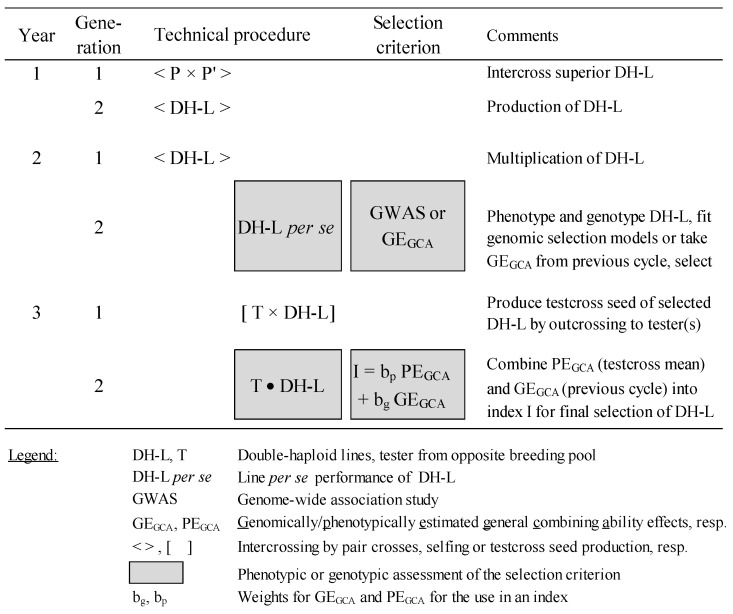
Technical description of selection scheme combining phenotypic and genomic selection for quantitative FSR resistance (adapted after [156]).

GS on the basis of DH lines only would be the most appropriate approach for improving FSR resistance (Figure 3) if the correlation between GCA effects and the performance of their inbred lines for FSR resistance is high enough.

The best DH lines will be selected using genomic selection (GS) in the first step of the (reciprocal) recurrent selection (RRS) scheme by first analyzing a genome-wide association study (GWAS) to develop a genomic model for FSR resistance. Due to the laborious and costly nature of the inoculation and rating systems, only moderate population sizes can be analyzed phenotypically by artificial infection. Based on these data, DH lines can be genomically selected and crossed afterward with a few testers (depending on the resource capacity of the breeder and the relative partitioning of GCA/SCA variances) from the opposite gene pool, undergoing a second step of RRS. The resulting testcrosses are evaluated phenotypically across several locations for their GCA effects. Mean phenotypic trait values and genomic estimated breeding values are used to select outstanding DH lines. In parallel, these data serve as a training population for the next cycle. The breeding program will benefit from GS in the future as the large effort in producing and phenotyping testcrosses for FSR resistance can be reserved for the putatively better part of the candidate DH population. Even more effective, a strong correlation between inbred lines and their hybrids in response to FSR resistance would allow for selection on the basis of the inbred line basis alone. The selection of testcrosses can be postponed to a later stage for grain yield evaluation. Then, a two-year scheme would then suffice for FSR improvement.

In large maize breeding companies, GS is routinely performed for traits like grain yield, dry matter yield, and maturity group, so there is no additional marker cost for GS for FSR resistance. Because of the additive inheritance of FSR resistance, both gene pools would have to go through the RRS and GS scheme, and resistance-selected DH lines from opposite gene pools can be used for creating new superior hybrid cultivars. Updating the GS model after introducing unrelated material is crucial. Combined-pool GS does not outperform within-pool GS regardless of the statistical model [89,104,157].

Previous studies have indicated that the incorporation of major QTLs as fixed effects can improve the performance of GS models. For example, studies on Gibberella ear rot resistance in maize showed GS weighted with significant QTLs to be about 20% more effective than MAS [104]. GS has successfully identified maize genotypes resistant to stalk rot by leveraging significant SNPs and accounting for genotype–environment interactions, increasing prediction accuracy in diverse populations [89]. For instance, the GS accuracy for the Tuxpeño population increased from 0.31 to 0.35, and for the non-Tuxpeño population from 0.60 to 0.70 by incorporating marker × environment information into the GS model [110]. These findings have shown the superiority of GS over MAS [89,110].

Key limitations of genomic selection (GS) are the need for constantly updating the training population and the need for a genetic relationship between the training and testing population. Incorporating unrelated populations may cause significant allele frequency fluctuations, further affecting GS effectiveness. Despite these restraints, GS remains a powerful tool for improving FSR resistance, accelerating genetic gain, shortening breeding cycles, capturing small-effect loci, and providing a cost-effective alternative to phenotyping.

## 5. Conclusions

Fusarium stalk rot (FSR) resistance is governed by a few major-effect QTLs and many minor-effect QTLs. It is crucial to verify these major QTLs across various maize populations to ensure their stability before they can be used in breeding. This verification aims to mitigate interactions between QTLs and genetic background, as well as interactions between QTLs and environmental factors [158]. In terms of phenotyping maize for FSR resistance, needle injection and a quantitative scale such as internode proportion are the most appropriate methods for plant breeders. Additionally, grain yield and high soluble sugar content can be included in a selection index for choosing FSR-resistant maize genotypes after physiological maturity. The moderate heritabilities for FSR resistance affords multi-locational testing of maize populations. Environmental variation coupled with low-throughput phenotyping further increases experimental error, reducing trait assessment accuracy and subsequently lowering prediction accuracy.

Although marker-assisted selection of known QTLs (*qRfg1*, *qRfg2*, *qRfg3*, and *Rgsr8.1*) can be utilized to breed resistant maize varieties, this method has drawbacks, including longer selection cycles compared to genomic selection (GS). GS can speed up breeding cycles, thereby increasing the annual genetic gain rate per unit of time [159]. The FSR-resistant hybrids should undergo a final evaluation with artificial infection in the field and additionally be assessed for mycotoxin content through immunotests to exclude those that do not meet safe mycotoxin levels. To enhance genetic diversity in FSR resistance, it would be beneficial to include landraces and germplasm resources from both tropical and temperate regions.

On a scientific basis, it could be productive to analyze the expression of possible candidate genes and their allelic variants and to elucidate the identity of the underlying resistance-causing factors. It would also be important to test the effect of these genes in different genetic backgrounds and in greenhouse as well as field environments.

## Figures and Tables

**Table 1:** Comparison of inoculation methods and disease ratings for assessing Fusarium stalk rot in maize.

Inoculation Method	*Fusarium* spp.	Inoculation Stage	Plant Material	Environments	Disease Rating	Reference
Needle injection	*F. graminearum*	V6 stage	67 I-lines	2	0–9 rating	[82]
	*F. graminearum*	VT stage	165 I-lines, F_2_ pop.	2	1–6 rating	[86]
	*F. graminearum*	V6 stage	97 I-lines	2	Disease severity index	[78]
	*F. graminearum*	VT stage	149 I-lines	2	Disease incidence	[87]
	*F. verticilliodes*	65 days after sowing	339 DH lines	3	1–9 rating	[88]
	*F. temperatum*	4-weeks old	7 cultivars	1	Hooker’s scale	[64]
Toothpick	*F. verticilliodes*	14 days after flowering	562 tropical I-lines	4	FSR severity (0–100%)	[89]
	*F. verticilliodes*	VT stage	342 tropical/subtropical I lines	4	Payak and Sharma scale	[90]
	*F. verticilliodes*	VT stage	54 I-lines, 3 hybrids, 1 OPV.	4	Hooker’s scale	[80]
Ball bearing pellet with a gas pistol	*F.* *verticilliodes*	7–10 days after anthesis	10 S_1_ lines	12	Hooker’s scale	[84]
Root infection	*F. graminearum*	Silk emergence	BC_1_F_1_, F_2_ pop.	1	1–9 rating	[91]
Oat kernels in internode	*F. graminearum*	12 days after flowering	6 hybrids, 12 parental lines	2	Colim 4.0 image analysis software	[92]

Abbreviations: I = inbred; VT = tasseling stage; OPV = open-pollinated varieties; V6 = six-leaf stage; pop. = population; DH = doubled haploid lines.

**Table 2:** Hooker’s quantitative ordinal scale for Fusarium stalk rot disease rating [100].

Disease Score	Degree of Infection	Disease Reaction
1	0 to 25% rot at the site of the inoculated internode	Highly resistant
2	26 to 50% rot of the inoculated internode	Resistant
3	51 to 75% rot of the inoculated internode	Moderately resistant
4	76 to 100% rot of the inoculated internode	Moderately susceptible
5	100% rot with infection extending into an adjacent internode	Susceptible

**Table 3:** Broad-sense heritability (*H*^2^) estimates of FSR resistance from different maize populations with reliable size.

*Fusarium* Species Target Region	Material	Pop. Size	No. Environments	*H* ^2^	Reference
*Fusarium graminearum*:					
Temperate	F_2:3_-L	150	1	0.37	[113]
	F_5:6_-L	199	3	0.86	[81]
Temperate and tropical	I-lines F_2_-L	165 350	2	0.60	[86]
*F. graminearum* and *F. verticilliodes*:					
Tropical	Tuxpeño Non-Tuxpeño	381 296	3	0.51 0.76	[110]
*F. verticilliodes:*					
Tropical	CML lines DTMA lines	280 282	4	0.77 0.55	[89]
	I-lines FSR-1 F_2:3_-L FSR-2 F_2:3_-L	342 256 166	4	0.51 0.40 0.48	[90]

Abbreviations: I = inbred; L = line; Pop. = population.

**Table 4:** Major named quantitative trait loci for Fusarium stalk rot.

Name	Chromosome	Bins	PVE (%)	Reference
*qRfg1*	10	10.03/4	36.3	[95]
*qRfg2*	1	1.09/10	8.9	[95]
*Rgsr8.1*	8	8.06/8	34.4	[91]
*qRfg3*	3	3.06/7	10.7–19.4	[81]

PVE = phenotypic variance explained by QTL.

## Data Availability

No new data are generated in this study. Data sharing is not applicable to this article.

## Supplementary Materials

The following supporting information can be downloaded at https://www.mdpi.com/article/10.3390/plants14050819/s1. Table S1: Details of studies for mapped quantitative trait loci (QTLs) and candidate genes for Fusarium stalk rot resistance in maize.
